# A monoclinic polymorph of (*R*,*R*)-4,4′-dibromo-2,2′-[cyclo­hexane-1,2-diylbis(nitrilo­methanylyl­idene)]diphenol

**DOI:** 10.1107/S1600536812016376

**Published:** 2012-04-21

**Authors:** Kwang Ha

**Affiliations:** a School of Applied Chemical Engineering, The Research Institute of Catalysis, Chonnam National University, Gwangju 500-757, Republic of Korea

## Abstract

The title compound, C_20_H_20_Br_2_N_2_O_2_, a tetra­dentate Schiff base, is the enanti­omerically pure *R*,*R*-diastereomer of four possible stereoisomers. The mol­ecular structure reveals two strong intra­molecular O—H⋯N hydrogen bonds between the hy­droxy O atom and the imino N atom, which each generate *S*(6) rings. In the crystal, mol­ecules are stacked in columns along the *a* axis; when viewed down the *b* axis, successive columns are stacked in the opposite direction. The structure reported herein is the monoclinic polymorph of the previously reported ortho­rhom­bic form [Yi & Hu (2009 ▶). *Acta Cryst*. E**65**, o2643], in which the complete mol­ecule is generated by a crystallographic twofold axis.

## Related literature

For the ortho­rhom­bic polymorph, see: Yi & Hu (2009 ▶).
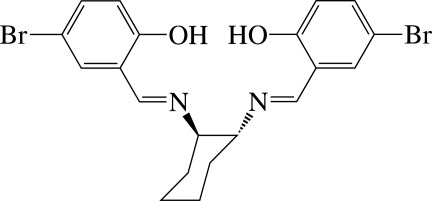


## Experimental

### 

#### Crystal data

C_20_H_20_Br_2_N_2_O_2_
*M* *_r_* = 480.20
Monoclinic, 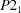

*a* = 5.9082 (5) Å
*b* = 18.8626 (15) Å
*c* = 9.0088 (7) Å
β = 91.867 (2)°
*V* = 1003.44 (14) Å^3^
*Z* = 2
Mo *K*α radiation
μ = 4.06 mm^−1^
*T* = 200 K
0.31 × 0.17 × 0.16 mm

#### Data collection

Bruker SMART 1000 CCD diffractometer
Absorption correction: multi-scan (*SADABS*; Bruker, 2000 ▶) *T*_min_ = 0.786, *T*_max_ = 1.000
7343 measured reflections
3868 independent reflections
2484 reflections with *I* > 2σ(*I*)
*R*_int_ = 0.031

#### Refinement

*R*[*F* ^2^ > 2σ(*F* ^2^)] = 0.039
*wR*(*F* ^2^) = 0.108
*S* = 1.03
3868 reflections
235 parameters
1 restraint
H-atom parameters constrained
Δρ_max_ = 0.88 e Å^−3^
Δρ_min_ = −0.47 e Å^−3^
Absolute structure: Flack (1983 ▶), 1331 Friedel pairs
Flack parameter: −0.010 (16)

### 

Data collection: *SMART* (Bruker, 2000 ▶); cell refinement: *SAINT* (Bruker, 2000 ▶); data reduction: *SAINT*; program(s) used to solve structure: *SHELXS97* (Sheldrick, 2008 ▶); program(s) used to refine structure: *SHELXL97* (Sheldrick, 2008 ▶); molecular graphics: *ORTEP-3* (Farrugia, 1997 ▶) and *PLATON* (Spek, 2009 ▶); software used to prepare material for publication: *SHELXL97*.

## Supplementary Material

Crystal structure: contains datablock(s) global, I. DOI: 10.1107/S1600536812016376/hb6734sup1.cif

Structure factors: contains datablock(s) I. DOI: 10.1107/S1600536812016376/hb6734Isup2.hkl

Additional supplementary materials:  crystallographic information; 3D view; checkCIF report

## Figures and Tables

**Table 1:** Hydrogen-bond geometry (Å, °)

*D*—H⋯*A*	*D*—H	H⋯*A*	*D*⋯*A*	*D*—H⋯*A*
O1—H1*O*⋯N1	0.84	1.82	2.581 (7)	150
O2—H2*O*⋯N2	0.84	1.87	2.626 (7)	149

**Fig. 1.**
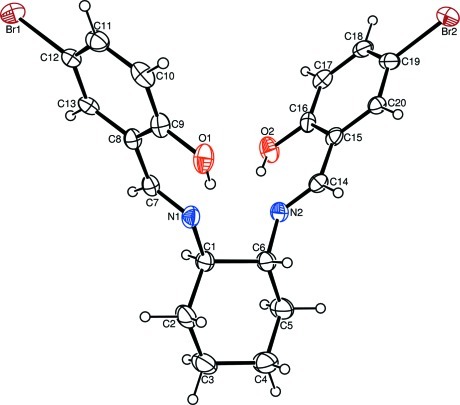
A structure detail of the title compound, with atom numbering. Displacement ellipsoids are drawn at the 30% probability level for non-H atoms.

**Fig. 2.**
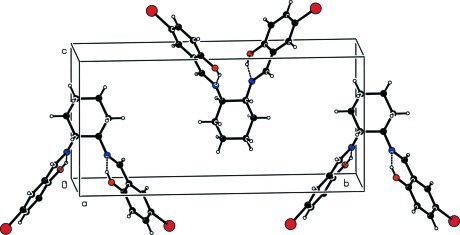
A view of the unit-cell contents of the title compound. Intramolecular O—H···N hydrogen-bond interactions are drawn with dashed lines.

## supplementary crystallographic information

### Comment

The crystal structure of the title compound, C_20_H_20_Br_2_N_2_O_2_, was previously reported in the orthorhombic space group *P*2_1_2_1_2 (Yi & Hu, 2009). The structure presented herein is essentially the same as the published structure and represents a monoclinic polymorph.

The title compound is a tetradentate Schiff base (Fig. 1), which can act as a dibasic ligand, *i.e.* the N and O donor atoms can coordinate one metal ion. The compound has two chiral C centres and is one of four possible stereoisomers. Crystallographically, the absolute configuration has been established by anomalous dispersion effects, and the *R* configuration of the asymmetric C atoms (C1 and C6) could be assigned. The Schiff base reveals strong intramolecular O—H···N hydrogen bonds between the hydroxy O atom and the imino N atom, with O···N distances of 2.581 (7) and 2.626 (7) Å, forming nearly planar six-membered rings (Fig. 2, Table 1). In the crystal structure, the benzene rings are not parallel: the dihedral angle between the benzene rings is 67.20 (15)°. The N—C bond lengths and the C—N—C bond angles indicate that the imino N atoms are *sp*^2^-hybridized [N1═C7 = 1.274 (8) Å, N1—C1 = 1.461 (8) Å, <C7—N1—C1 = 118.7 (6)°; N2═C14 = 1.280 (8) Å, N2—C6 = 1.474 (8) Å, <C14—N2—C6 = 118.3 (6)°]. The molecules are stacked in columns along the *a* axis. When viewed down the *b* axis, the successive compounds are stacked in the opposite direction. In the columns, the shortest centroid-centroid distance between aromatic rings is 4.709 (3) Å.

### Experimental

1,2-Diaminocyclohexane (0.8007 g, 7.012 mmol) and 5-bromosalicylaldehyde (2.8204 g, 14.031 mmol) in EtOH (20 ml) were stirred for 1 h at room temperature. After addition of pentane (30 ml) to the reaction mixture, the formed precipitate was separated by filtration, washed with ether, and dried at 323 K, to give a yellow powder (1.7660 g). Yellow blocks were obtained by slow evaporation from a CH_3_CN solution at room temperature. The previous polymorph (Yi & Hu, 2009) was crystallised from methanol.

### Refinement

Carbon-bound H atoms were positioned geometrically and allowed to ride on their respective parent atoms: C—H = 0.95–1.00 Å with *U*_iso_(H) = 1.2*U*_eq_(C). The hydroxy H atoms were located from the difference Fourier map then allowed to ride on their parent atoms in the final cycles of refinement with O—H = 0.84 Å and *U*_iso_(H) = 1.5 *U*_eq_(O). The highest peak (0.88 e Å^-3^) and the deepest hole (-0.47 e Å^-3^) in the difference Fourier map are located 1.34 Å and 0.86 Å, respectively, from the atoms Br1 and Br2. The absolute configuration was established by anomalous dispersion effects *via* diffraction measurements on the crystal. The Flack parameter is -0.010 (16) in the final cycles of refinement.

### Crystal data

**Table:**

C_20_H_20_Br_2_N_2_O_2_	*F*(000) = 480
*M**_r_* = 480.20	*D*_x_ = 1.589 Mg m^−^^3^
Monoclinic, *P*2_1_	Mo *K*α radiation, λ = 0.71073 Å
Hall symbol: P 2yb	Cell parameters from 2758 reflections
*a* = 5.9082 (5) Å	θ = 2.5–25.8°
*b* = 18.8626 (15) Å	µ = 4.06 mm^−^^1^
*c* = 9.0088 (7) Å	*T* = 200 K
β = 91.867 (2)°	Block, yellow
*V* = 1003.44 (14) Å^3^	0.31 × 0.17 × 0.16 mm
*Z* = 2	

### Data collection

**Table:**

Bruker SMART 1000 CCD diffractometer	3868 independent reflections
Radiation source: fine-focus sealed tube	2484 reflections with *I* > 2σ(*I*)
Graphite monochromator	*R*_int_ = 0.031
φ and ω scans	θ_max_ = 28.3°, θ_min_ = 2.2°
Absorption correction: multi-scan (*SADABS*; Bruker, 2000)	*h* = −7→7
*T*_min_ = 0.786, *T*_max_ = 1.000	*k* = −20→25
7343 measured reflections	*l* = −11→11

### Refinement

**Table:**

Refinement on *F*^2^	Secondary atom site location: difference Fourier map
Least-squares matrix: full	Hydrogen site location: inferred from neighbouring sites
*R*[*F*^2^ > 2σ(*F*^2^)] = 0.039	H-atom parameters constrained
*wR*(*F*^2^) = 0.108	*w* = 1/[σ^2^(*F*_o_^2^) + (0.0434*P*)^2^] where *P* = (*F*_o_^2^ + 2*F*_c_^2^)/3
*S* = 1.03	(Δ/σ)_max_ < 0.001
3868 reflections	Δρ_max_ = 0.88 e Å^−^^3^
235 parameters	Δρ_min_ = −0.47 e Å^−^^3^
1 restraint	Absolute structure: Flack (1983), 1331 Friedel pairs
Primary atom site location: structure-invariant direct methods	Flack parameter: −0.010 (16)

### Special details

**Table:**

Geometry. All e.s.d.'s (except the e.s.d. in the dihedral angle between two l.s. planes) are estimated using the full covariance matrix. The cell e.s.d.'s are taken into account individually in the estimation of e.s.d.'s in distances, angles and torsion angles; correlations between e.s.d.'s in cell parameters are only used when they are defined by crystal symmetry. An approximate (isotropic) treatment of cell e.s.d.'s is used for estimating e.s.d.'s involving l.s. planes.
Refinement. Refinement of *F*^2^ against ALL reflections. The weighted *R*-factor *wR* and goodness of fit *S* are based on *F*^2^, conventional *R*-factors *R* are based on *F*, with *F* set to zero for negative *F*^2^. The threshold expression of *F*^2^ > σ(*F*^2^) is used only for calculating *R*-factors(gt) *etc*. and is not relevant to the choice of reflections for refinement. *R*-factors based on *F*^2^ are statistically about twice as large as those based on *F*, and *R*- factors based on ALL data will be even larger.

### Fractional atomic coordinates and isotropic or equivalent isotropic displacement parameters (Å^2^)

**Table:**

	*x*	*y*	*z*	*U*_iso_*/*U*_eq_	
Br1	0.25215 (12)	0.33723 (3)	−0.33161 (7)	0.0600 (3)	
Br2	0.27364 (11)	−0.23777 (3)	−0.33104 (7)	0.0551 (2)	
O1	0.7141 (7)	0.1204 (3)	0.0604 (6)	0.0539 (13)	
H1O	0.6245	0.1070	0.1252	0.081*	
O2	−0.2482 (8)	−0.0312 (3)	0.0322 (6)	0.0587 (14)	
H2O	−0.1588	−0.0127	0.0955	0.088*	
N1	0.3538 (9)	0.1159 (3)	0.2147 (5)	0.0428 (12)	
N2	0.1109 (9)	−0.0185 (2)	0.2097 (5)	0.0427 (12)	
C1	0.2247 (11)	0.0899 (5)	0.3390 (7)	0.0414 (18)	
H1	0.0634	0.1054	0.3257	0.050*	
C2	0.3242 (15)	0.1210 (4)	0.4830 (7)	0.0601 (19)	
H2A	0.4888	0.1111	0.4893	0.072*	
H2B	0.3038	0.1731	0.4816	0.072*	
C3	0.2143 (15)	0.0908 (5)	0.6200 (10)	0.068 (2)	
H3A	0.2893	0.1107	0.7106	0.081*	
H3B	0.0525	0.1045	0.6194	0.081*	
C4	0.2344 (16)	0.0103 (5)	0.6219 (10)	0.071 (3)	
H4A	0.1588	−0.0089	0.7097	0.086*	
H4B	0.3961	−0.0035	0.6288	0.086*	
C5	0.1241 (15)	−0.0204 (4)	0.4802 (7)	0.061 (2)	
H5A	0.1385	−0.0727	0.4818	0.073*	
H5B	−0.0392	−0.0085	0.4763	0.073*	
C6	0.2343 (11)	0.0086 (5)	0.3428 (8)	0.0452 (19)	
H6	0.3957	−0.0073	0.3416	0.054*	
C7	0.2650 (10)	0.1629 (4)	0.1295 (7)	0.0373 (15)	
H7	0.1164	0.1795	0.1471	0.045*	
C8	0.3885 (9)	0.1916 (3)	0.0051 (6)	0.0344 (12)	
C9	0.6051 (10)	0.1676 (3)	−0.0268 (7)	0.0439 (14)	
C10	0.7125 (11)	0.1940 (5)	−0.1518 (7)	0.0477 (19)	
H10	0.8586	0.1771	−0.1751	0.057*	
C11	0.6082 (11)	0.2443 (3)	−0.2412 (6)	0.0459 (15)	
H11	0.6819	0.2623	−0.3255	0.055*	
C12	0.3947 (9)	0.2685 (3)	−0.2071 (5)	0.0406 (12)	
C13	0.2877 (10)	0.2432 (3)	−0.0852 (7)	0.0381 (16)	
H13	0.1428	0.2612	−0.0622	0.046*	
C14	0.2091 (10)	−0.0645 (4)	0.1293 (7)	0.0378 (15)	
H14	0.3597	−0.0784	0.1555	0.045*	
C15	0.0974 (10)	−0.0959 (3)	−0.0004 (6)	0.0366 (13)	
C16	−0.1247 (10)	−0.0773 (3)	−0.0454 (6)	0.0387 (13)	
C17	−0.2241 (12)	−0.1068 (4)	−0.1734 (7)	0.0443 (18)	
H17	−0.3742	−0.0939	−0.2032	0.053*	
C18	−0.1054 (10)	−0.1550 (3)	−0.2579 (6)	0.0443 (14)	
H18	−0.1728	−0.1752	−0.3452	0.053*	
C19	0.1098 (10)	−0.1725 (3)	−0.2129 (5)	0.0416 (13)	
C20	0.2136 (9)	−0.1450 (3)	−0.0861 (6)	0.0333 (14)	
H20	0.3628	−0.1592	−0.0572	0.040*	

### Atomic displacement parameters (Å^2^)

**Table:**

	*U*^11^	*U*^22^	*U*^33^	*U*^12^	*U*^13^	*U*^23^
Br1	0.0820 (6)	0.0589 (6)	0.0384 (4)	0.0047 (4)	−0.0070 (3)	0.0073 (4)
Br2	0.0668 (5)	0.0587 (6)	0.0400 (4)	0.0071 (4)	0.0051 (3)	−0.0076 (4)
O1	0.042 (3)	0.041 (3)	0.080 (4)	0.010 (2)	0.004 (2)	0.005 (3)
O2	0.044 (3)	0.050 (3)	0.082 (4)	0.010 (2)	0.003 (3)	−0.012 (3)
N1	0.049 (3)	0.030 (3)	0.049 (3)	−0.004 (2)	0.000 (2)	0.002 (2)
N2	0.056 (3)	0.031 (3)	0.042 (3)	−0.007 (2)	0.003 (2)	0.000 (2)
C1	0.049 (4)	0.033 (4)	0.042 (5)	−0.007 (3)	0.004 (3)	0.000 (3)
C2	0.089 (6)	0.039 (4)	0.053 (4)	−0.013 (4)	0.007 (4)	−0.013 (3)
C3	0.100 (7)	0.058 (6)	0.046 (5)	−0.023 (5)	0.006 (4)	−0.014 (5)
C4	0.111 (7)	0.056 (6)	0.046 (5)	−0.022 (5)	−0.001 (4)	0.003 (5)
C5	0.086 (6)	0.047 (4)	0.049 (4)	−0.026 (4)	0.003 (4)	−0.004 (3)
C6	0.060 (5)	0.035 (5)	0.041 (5)	−0.012 (3)	−0.001 (4)	−0.002 (3)
C7	0.036 (3)	0.030 (4)	0.045 (4)	−0.001 (3)	−0.002 (3)	−0.007 (3)
C8	0.031 (3)	0.030 (3)	0.041 (3)	0.000 (2)	−0.002 (2)	−0.003 (2)
C9	0.041 (3)	0.035 (3)	0.056 (4)	0.004 (3)	0.000 (3)	−0.004 (3)
C10	0.038 (3)	0.048 (5)	0.058 (4)	−0.008 (3)	0.011 (3)	−0.018 (3)
C11	0.051 (4)	0.047 (4)	0.040 (3)	−0.011 (3)	0.007 (3)	−0.007 (3)
C12	0.054 (3)	0.036 (3)	0.031 (3)	0.000 (3)	−0.001 (2)	−0.006 (3)
C13	0.040 (3)	0.043 (4)	0.031 (3)	0.000 (3)	−0.001 (2)	−0.012 (3)
C14	0.038 (3)	0.038 (4)	0.038 (3)	−0.003 (3)	0.003 (3)	0.007 (3)
C15	0.042 (3)	0.029 (3)	0.038 (3)	−0.006 (2)	0.001 (2)	0.009 (2)
C16	0.039 (3)	0.029 (3)	0.049 (3)	0.004 (2)	0.006 (3)	0.004 (3)
C17	0.044 (4)	0.044 (4)	0.044 (4)	−0.003 (3)	−0.003 (3)	0.012 (3)
C18	0.056 (4)	0.044 (4)	0.032 (3)	−0.007 (3)	−0.005 (2)	0.007 (3)
C19	0.047 (3)	0.047 (4)	0.031 (3)	−0.002 (3)	0.002 (2)	0.000 (3)
C20	0.029 (3)	0.037 (4)	0.033 (3)	0.002 (2)	0.001 (2)	0.003 (3)

### Geometric parameters (Å, º)

**Table:**

Br1—C12	1.894 (6)	C5—H5B	0.9900
Br2—C19	1.912 (6)	C6—H6	1.0000
O1—C9	1.337 (7)	C7—C8	1.461 (8)
O1—H1O	0.8400	C7—H7	0.9500
O2—C16	1.345 (7)	C8—C13	1.391 (8)
O2—H2O	0.8400	C8—C9	1.396 (8)
N1—C7	1.274 (8)	C9—C10	1.403 (9)
N1—C1	1.461 (8)	C10—C11	1.377 (10)
N2—C14	1.280 (8)	C10—H10	0.9500
N2—C6	1.474 (8)	C11—C12	1.385 (8)
C1—C2	1.524 (10)	C11—H11	0.9500
C1—C6	1.534 (7)	C12—C13	1.370 (8)
C1—H1	1.0000	C13—H13	0.9500
C2—C3	1.523 (10)	C14—C15	1.451 (9)
C2—H2A	0.9900	C14—H14	0.9500
C2—H2B	0.9900	C15—C20	1.398 (8)
C3—C4	1.522 (8)	C15—C16	1.405 (8)
C3—H3A	0.9900	C16—C17	1.393 (9)
C3—H3B	0.9900	C17—C18	1.390 (9)
C4—C5	1.528 (11)	C17—H17	0.9500
C4—H4A	0.9900	C18—C19	1.363 (8)
C4—H4B	0.9900	C18—H18	0.9500
C5—C6	1.520 (10)	C19—C20	1.380 (8)
C5—H5A	0.9900	C20—H20	0.9500
			
C9—O1—H1O	107.9	N1—C7—H7	119.6
C16—O2—H2O	106.3	C8—C7—H7	119.6
C7—N1—C1	118.7 (6)	C13—C8—C9	119.0 (5)
C14—N2—C6	118.3 (6)	C13—C8—C7	119.6 (5)
N1—C1—C2	109.1 (6)	C9—C8—C7	121.5 (5)
N1—C1—C6	109.5 (7)	O1—C9—C8	121.4 (5)
C2—C1—C6	110.6 (7)	O1—C9—C10	119.1 (6)
N1—C1—H1	109.2	C8—C9—C10	119.4 (6)
C2—C1—H1	109.2	C11—C10—C9	120.6 (6)
C6—C1—H1	109.2	C11—C10—H10	119.7
C3—C2—C1	112.5 (7)	C9—C10—H10	119.7
C3—C2—H2A	109.1	C10—C11—C12	119.4 (6)
C1—C2—H2A	109.1	C10—C11—H11	120.3
C3—C2—H2B	109.1	C12—C11—H11	120.3
C1—C2—H2B	109.1	C13—C12—C11	120.7 (5)
H2A—C2—H2B	107.8	C13—C12—Br1	120.3 (4)
C4—C3—C2	110.3 (8)	C11—C12—Br1	119.0 (4)
C4—C3—H3A	109.6	C12—C13—C8	120.9 (5)
C2—C3—H3A	109.6	C12—C13—H13	119.6
C4—C3—H3B	109.6	C8—C13—H13	119.6
C2—C3—H3B	109.6	N2—C14—C15	122.0 (6)
H3A—C3—H3B	108.1	N2—C14—H14	119.0
C3—C4—C5	109.8 (9)	C15—C14—H14	119.0
C3—C4—H4A	109.7	C20—C15—C16	118.5 (5)
C5—C4—H4A	109.7	C20—C15—C14	119.8 (5)
C3—C4—H4B	109.7	C16—C15—C14	121.7 (5)
C5—C4—H4B	109.7	O2—C16—C17	117.8 (6)
H4A—C4—H4B	108.2	O2—C16—C15	122.0 (5)
C6—C5—C4	111.2 (6)	C17—C16—C15	120.2 (6)
C6—C5—H5A	109.4	C18—C17—C16	120.5 (6)
C4—C5—H5A	109.4	C18—C17—H17	119.7
C6—C5—H5B	109.4	C16—C17—H17	119.7
C4—C5—H5B	109.4	C19—C18—C17	118.5 (6)
H5A—C5—H5B	108.0	C19—C18—H18	120.7
N2—C6—C5	108.9 (6)	C17—C18—H18	120.7
N2—C6—C1	108.2 (7)	C18—C19—C20	122.7 (5)
C5—C6—C1	111.2 (7)	C18—C19—Br2	118.3 (4)
N2—C6—H6	109.5	C20—C19—Br2	119.0 (4)
C5—C6—H6	109.5	C19—C20—C15	119.5 (5)
C1—C6—H6	109.5	C19—C20—H20	120.2
N1—C7—C8	120.9 (6)	C15—C20—H20	120.2
			
C7—N1—C1—C2	−106.0 (7)	C9—C10—C11—C12	−0.3 (10)
C7—N1—C1—C6	132.8 (6)	C10—C11—C12—C13	0.4 (9)
N1—C1—C2—C3	−174.3 (7)	C10—C11—C12—Br1	−179.6 (5)
C6—C1—C2—C3	−53.9 (9)	C11—C12—C13—C8	−1.4 (9)
C1—C2—C3—C4	56.4 (11)	Br1—C12—C13—C8	178.5 (4)
C2—C3—C4—C5	−57.8 (10)	C9—C8—C13—C12	2.4 (9)
C3—C4—C5—C6	58.7 (10)	C7—C8—C13—C12	−176.7 (5)
C14—N2—C6—C5	−109.5 (7)	C6—N2—C14—C15	177.7 (6)
C14—N2—C6—C1	129.6 (7)	N2—C14—C15—C20	178.8 (6)
C4—C5—C6—N2	−175.8 (7)	N2—C14—C15—C16	0.4 (9)
C4—C5—C6—C1	−56.7 (9)	C20—C15—C16—O2	179.1 (5)
N1—C1—C6—N2	−66.8 (7)	C14—C15—C16—O2	−2.4 (8)
C2—C1—C6—N2	173.0 (5)	C20—C15—C16—C17	−0.6 (9)
N1—C1—C6—C5	173.7 (5)	C14—C15—C16—C17	177.9 (6)
C2—C1—C6—C5	53.5 (8)	O2—C16—C17—C18	−179.7 (6)
C1—N1—C7—C8	178.9 (6)	C15—C16—C17—C18	0.1 (10)
N1—C7—C8—C13	−179.5 (6)	C16—C17—C18—C19	−0.1 (10)
N1—C7—C8—C9	1.5 (9)	C17—C18—C19—C20	0.7 (9)
C13—C8—C9—O1	176.9 (5)	C17—C18—C19—Br2	−179.2 (5)
C7—C8—C9—O1	−4.1 (9)	C18—C19—C20—C15	−1.3 (9)
C13—C8—C9—C10	−2.3 (9)	Br2—C19—C20—C15	178.6 (4)
C7—C8—C9—C10	176.7 (5)	C16—C15—C20—C19	1.2 (8)
O1—C9—C10—C11	−177.9 (6)	C14—C15—C20—C19	−177.3 (5)
C8—C9—C10—C11	1.3 (10)		

### Hydrogen-bond geometry (Å, º)

**Table:**

*D*—H···*A*	*D*—H	H···*A*	*D*···*A*	*D*—H···*A*
O1—H1*O*···N1	0.84	1.82	2.581 (7)	150
O2—H2*O*···N2	0.84	1.87	2.626 (7)	149
